# Petroleum Contamination and Plant Identity Influence Soil and Root Microbial Communities While AMF Spores Retrieved from the Same Plants Possess Markedly Different Communities

**DOI:** 10.3389/fpls.2017.01381

**Published:** 2017-08-08

**Authors:** Bachir Iffis, Marc St-Arnaud, Mohamed Hijri

**Affiliations:** Institut de Recherche en Biologie Végétale, Université de Montréal, Montréal QC, Canada

**Keywords:** petroleum hydrocarbon pollutants, 454 high-throughput sequencing, phytoremediation, arbuscular mycorrhizal fungi, bacteria, microbial ecology, plant–microbe interaction

## Abstract

Phytoremediation is a promising *in situ* green technology based on the use of plants to cleanup soils from organic and inorganic pollutants. Microbes, particularly bacteria and fungi, that closely interact with plant roots play key roles in phytoremediation processes. In polluted soils, the root-associated microbes contribute to alleviation of plant stress, improve nutrient uptake and may either degrade or sequester a large range of soil pollutants. Therefore, improving the efficiency of phytoremediation requires a thorough knowledge of the microbial diversity living in the rhizosphere and in close association with plant roots in both the surface and the endosphere. This study aims to assess fungal ITS and bacterial 16S rRNA gene diversity using high-throughput sequencing in rhizospheric soils and roots of three plant species (*Solidago canadensis, Populus balsamifera*, and *Lycopus europaeus*) growing spontaneously in three petroleum hydrocarbon polluted sedimentation basins. Microbial community structures of rhizospheric soils and roots were compared with those of microbes associated with arbuscular mycorrhizal fungal (AMF) spores to determine the links between the root and rhizosphere communities and those associated with AMF. Our results showed a difference in OTU richness and community structure composition between soils and roots for both bacteria and fungi. We found that petroleum hydrocarbon pollutant (PHP) concentrations have a significant effect on fungal and bacterial community structures in both soils and roots, whereas plant species identity showed a significant effect only on the roots for bacteria and fungi. Our results also showed that the community composition of bacteria and fungi in soil and roots varied from those associated with AMF spores harvested from the same plants. This let us to speculate that in petroleum hydrocarbon contaminated soils, AMF may release chemical compounds by which they recruit beneficial microbes to tolerate or degrade the PHPs present in the soil.

## Introduction

During the past century, industrial production, urbanization, energy consumption, transportation and human population have expanded exponentially, resulting in increased soil, water and air pollution, which in turn has placed the environment under substantial pressure (Samanta et al., 2002; Chen and Kan, 2008). Together, these factors produced a large number of highly polluted sites all over the planet, usually containing complex mixtures of toxic and carcinogenic, organic and inorganic compounds. Organic contaminants such as polycyclic aromatic hydrocarbons (PAH) are known mutagens and carcinogens that enter the food chain together with lipophilic compounds (Boffetta et al., 1997; Henner et al., 1997; Poirier, 2004). Inorganic contaminants mainly consist of metalloids and trace metals with soil retention times of up to thousands of years. Like organic compounds, they reduce plant growth, negatively impact the soil microbiota (McGrath et al., 2001), and decrease the quality of the environment (Gremion et al., 2004) to such an extent that they pose serious health risks to humans and animals.

Polluted sites may be cleaned by physico-chemical strategies including excavation and storage, washing, and chemical treatments. Yet, most *ex situ* treatments only contain contamination without eliminating it. They damage or even destroy soil microbial communities, and are unfit for application over large areas because they are prohibitively expensive. An alternative, most promising *in situ* approach for multi-contaminated sites is phytoremediation (Salt et al., 1995, 1998; Peuke and Rennenberg, 2005), which uses plants and their associated soil microbial communities (fungi and bacteria) to accumulate pollutants within the plants, and/or degrade them in the soil (Peuke and Rennenberg, 2005; Pilon-Smits, 2005; Bieby Voijant et al., 2011).

In the past few years, phytoremediation has become increasingly popular owing to its efficiency, cost effectiveness and respect for the integrity of the soil structure and biology. However, in most cases, phytoremediation is achieved through a complex interaction between plants and the myriad of bacteria and fungi living in the rhizospheric soil and in association with plant roots (endophytic and epiphytic microorganisms) (Pilon-Smits, 2005; Jing et al., 2007; Bourdel et al., 2016; Rohrbacher and St-Arnaud, 2016; Thijs et al., 2016). Therefore, improving the efficiency of phytoremediation requires a thorough knowledge of the microbial diversity living in the rhizospheric soil and in association with plant roots (either in the rhizosphere or the endosphere), and of their interactions with plants. Several studies demonstrated that, in contaminated soils, the root exudates released in the plant rhizosphere promote the selection of microorganisms able to degrade pollutants and stimulate the expression of several genes involved in xenobiotic compound degradation (Bell et al., 2014; Yergeau et al., 2014; Pagé et al., 2015; Rohrbacher and St-Arnaud, 2016; Thijs et al., 2016). For example, Pagé et al. (2015) used a metatranscriptomic approach to compare the gene expression of 10 oxygenases related to petroleum hydrocarbon degradation between the bulk and rhizospheric soils of *Salix purpurea.* They found that among the 10 genes examined, four of them were significantly over-expressed in rhizospheric soil compared with bulk soil. Bell et al. (2014) studied the fungal and bacterial diversity from the rhizospheric soils of eleven cultivars of willow planted in three hydrocarbon contaminated sites, and they found that the abundance of some petroleum hydrocarbon-degrading microorganisms, such as some classes of *Proteobacteria* (*Alpha-, Beta-*, and *Gamma-Proteobacteria*) and *Dothideomycetes* (fungi), were significantly enhanced in highly contaminated (HC) plots compared with the low and non-contaminated plots.

Arbuscular mycorrhizal fungi (AMF) are well known as soil fungi able to establish a mutualistic symbiosis with most of land plants (Smith and Read, 2008). In exchange for carbon resources, AMF provide the host plants with nutrients and protect them against soil-borne pathogens (St-Arnaud and Vujanovic, 2007; Smith and Read, 2008; Ismail et al., 2011). In addition, many reports have shown that AMF may play an important role in soil phytoremediation processes (Liu and Dalpé, 2009; Wu et al., 2009; Gao et al., 2011; Hassan et al., 2013). In the soil surrounding plant roots, AMF share the same micro-environment with other rhizospheric microorganisms and several studies have suggested that AMF species collaborate with some of these microorganisms in phytoremediation process (Alarcón et al., 2008; Liu and Dalpé, 2009; Teng et al., 2010). However, AMF also harbor their own hyphosphere microorganisms on the surface of their spores and mycelia (Hijri et al., 2002; Bonfante and Anca, 2009; Scheublin et al., 2010; Lecomte et al., 2011), and the role of these microbes in phytoremediation processes is unknown.

Using 454 sequencing, Iffis et al. (2016) have conducted a study of bacteria and fungi associated to AMF spores harvested from soil collected from the rooting zone of three plant species (*Solidago canadensis, Populus balsamifera*, and *Lycopus europaeus*) growing spontaneously in waste decantation basins of a former petrochemical plant. They have found a large diversity of bacteria and fungi in association with the AMF spores. They also found that the AMF-associated fungal and bacterial communities were significantly affected by both petroleum hydrocarbon pollutant (PHP) concentrations and plant species identity. Furthermore, Iffis et al. (2016) observed that some AMF taxa were either positively or negatively correlated with some fungal and bacterial groups, suggesting that AMF may also play a role in shaping the microbial communities associated with their spores. Similarly, Iffis et al. (2014) showed that the intraradical propagules (vesicules and spores inside plant roots) of AMF extracted from *S. rugosa* roots, sampled in a PHP contaminated site, also harbored a large diversity of bacteria and fungi.

Based on these studies, we inferred that plant species, AMF community and PHP concentrations are among the major driving forces that shape microbial communities living in the rhizosphere and in association with the roots of plants growing in petroleum hydrocarbon polluted sites. The current study aims to understand the contribution of each of these factors on bacterial and fungal community structures in rhizospheric soils and roots sampled in polluted sedimentation basins. Our objectives were: (i) to assess the bacterial and fungal diversity associated with roots and their surrounding soil from mycorrhizal plants spontaneously growing in waste decantation basins of a former petrochemical plant; (ii) to test the effects of PHP and plant species identity on the microbial community structure in the soil and plant roots; and (iii) to compare the microbial community structure of soil and roots with the microbial community structure associated with the AMF spores in order to verify the hypothesis that AMF are able to recruit specific microbial communities on the surface of their spores and mycelia.

To do so, soil and root samples of three plant species (*S. canadensis, P. balsamifera*, and *L. europaeus*) growing spontaneously in three petroleum hydrocarbon polluted sedimentation basins that were used in Iffis et al. (2016) to assess the AMF-spore associated microbes were subjected to DNA extraction, PCR amplifications targeting the ITS regions for fungi and 16S rRNA gene for bacteria, then the PCR products were sequenced using the 454 FLX+ high throughput sequencing platform to profile the microbial communities structure.

Overall, our results show a difference in OTU richness and community structure composition between soil and roots for both bacteria and fungi. We also found that PHP concentrations have a significant effect on the fungal and bacterial community structures in both soil and roots while, plant species identity had a significant effect only on the root bacteria and fungi. Furthermore, the comparison between the results of this study and Iffis et al. (2016) study showed that the microbial community structures found in soil and root differed from those found in association with the AMF spores harvested from the same samples. These results support the hypothesis that AMF can recruit specific microbial communities on the surface of their spores and mycelia. To our knowledge, this is the first research work devoted to compare the microbial communities of soil, roots and those associated with AMF spores in petroleum hydrocarbon contaminated sites.

## Materials and Methods

### Experimental Design and Sampling

Field sampling and experimental design were previously described in Iffis et al. (2016). Briefly, *S. canadensis, P. balsamifera*, and *L. europaeus* are three plant species naturally growing in three petroleum contaminated basins of a former petrochemical plant located on the south shore of the St-Lawrence River near Montreal, QC, Canada (45.70 N, 73.43 W) (Desjardins et al., 2014) (Supplementary Figure S1). *P. balsamifera* trees were between 0.5 and 1 m height and they were approximately one or 2 years old. The root system of three individual plants with their surrounding soils were collected for each plant species and from each basin, totalizing 27 samples (three basins × three plant species × three individual plants per species). Here, we define rhizosphere soil as soil surrounding roots and under influence of plant roots obtained by shaking the roots in a non-sterile plastic bag (Ziploc Freezer bags, dimensions: 26.8 cm × 27.3 cm) and collecting the remaining soil. Samples were immediately put and transported in a cooler filled with ice packs. Soils were homogenized by shaking bags. Soil subsamples were collected and stored at -20°C until use.

The root system from each plant was washed several times in tap water until elimination of all soils debris attached, and then cut into 1 cm long pieces followed by another round of wash by sterilized water. Since a part of the root samples was already used for microscopic examination, the remaining amounts were insufficient for individual DNA extractions. Therefore, the root replicates of each plant species per basin were pooled in 10 ml tubes, and ground in liquid nitrogen with a mortar and pestle. Then, 500 mg aliquots of the ground root material was collected in 1.5 ml tubes, totaling nine samples for plant roots. As the remaining soil amount was sufficient for individual DNA extraction, the soil samples were kept separate for each replicate per plant species from each basin, so there was a total of 27 soil samples. For each replicate, the soil surrounding the roots was collected in plastic bags, homogenized by shaking, then 500 mg aliquots of this soil were collected in 1.5 ml tubes for DNA extraction. Root and soil tubes were conserved at -20°C until use.

The analysis of PAH and total alkane (C10–C50) concentrations from the soil of the three contaminated basins was carried out using a commercial laboratory service (Maxxam Analytical Laboratories, Montreal, QC, Canada), and the results are available in Supplementary Table S10 of Iffis et al. (2016). Basin 1 was the most highly contaminated with mean concentration of alkanes (C10–C50) equal to 2200 mg/kg of soil (termed as HC), basin 2 was the least contaminated with 153 mg/kg of soil (termed as LC), while the basin 3 was moderately contaminated with 800 mg/kg of soil (termed as MC) (Iffis et al., 2016).

### DNA Extraction and Polymerase Chain Reactions

DNA extraction was performed in both soil and roots samples using the NucleoSpin Soil Kit (MACHEREY-NAGEL, United States) following the manufacturer’s instructions. DNA was eluted in a 50 μl of elution buffer, and was stored at -20°C until use.

To identify the bacterial taxa, soil and root DNA samples were subjected to PCR amplification targeting the V1–V4 hypervariable region of 16S rRNA gene with primer pair UnivBactF 9 (5′GAGTTTGATYMTGGCTC-3′) and BSR534/18 (5′-ATTACCGCGGCTGCTGGC-3′). We choose these primers because they were successfully used and tested in 454 pyrosequencing by Bell et al. (2011, 2014) and Yergeau et al. (2012). To identify the fungal taxa, the ITS regions were targeted using primer pair ITS1F and ITS4 (Bell et al., 2014). For soil DNA samples, PCR amplifications were performed once on each DNA sample, totaling 27 PCR tubes of soil samples. For root DNA, since plant root replicates were pooled within each plant species per basin, PCR amplifications were performed in triplicate on each DNA sample, also totaling 27 PCRs tubes.

Both 16S and ITS primers were tagged with adaptors and unique multiplex identifier (MID) tags from the extended MID set recommended by Roche Diagnostics (Roche, 2009). PCRs were performed in 50 μl volumes containing 5 μl of 10× PCR buffer, 0.2 mM of dNTP mix, 1 μl of BSA (100 mg/ml), 1 μl MgCl_2_ (25mM), 0.4 mM of each primer, 2 μl of DNA template and 1 U of *Taq* DNA polymerase (QIAGEN, Toronto, ON, Canada). PCRs were run on a thermal cycler Pro S thermocycler (Eppendorf, Mississauga, ON, Canada) using an initial denaturation at 95°C for 5 min followed by 35 cycles of 94°C for 30 s, 55°C for 30 s, 72°C for 1 min, and a final elongation step at 72°C for 10 min. After electrophoresis separation and UV light visualization, the PCRs products were purified with the QIAquick Gel Extraction Kit (QIAGEN, Toronto, ON, Canada) following the manufacturer’s instructions. Then, DNA concentrations of the purified products were measured using the Qubit 2.0 fluorometer (Life Technologies, Burlington, ON, Canada). Four pools were prepared by mixing equimolar volumes of PCR products. The four pools were 16S rRNA gene amplicons of soil samples, 16S rRNA amplicons of root samples, ITS amplicons of soil samples and ITS amplicons of root samples. These pools were sent for sequencing to the McGill University and Genome Québec Innovation Centre using the Roche 454 FLX+ pyrosequencing platform (Roche, Branford, CT, United States), with one-eighth of sequencing plate per pool.

The sequences generated in this study were deposited in the NCBI Sequence Read Archive and are available under the project number SRP100801.

### Bioinformatic Processing

Sequence processing was performed in Mothur (v.1.34.4) (Schloss et al., 2009) as described in the Mothur wiki^1^, with some minor modifications (Bell et al., 2015). Briefly, for both soil and root 16S rRNA gene sequences, the “.sff” files of the different samples were first merged in one “.sff” file, then “.qual,” and “.fasta” files were obtained from the “.sff” file using “merge.files” and “sffinfo” commands, respectively. Low quality and short sequences were removed using the “trim.seqs” command, with the following parameters: maxambig = 0, maxhomop = 8, bdiffs = 1, pdiffs = 2, qwindowaverage = 30, qwindowsize = 50, minlength = 300 and then, we reduced the dataset to only unique sequences using the “unique.seqs” command. After sequence alignments against the Mothur interpreted Silva bacterial database using “align.seqs,” the non-aligned sequences were removed using the “screen.seqs” command with the following criteria: start = 1044, optimize = end, criteria = 95. Prior to sequence classifications, the datasets were first subjected to a second simplification using the “unique.seq” command, followed by the commands “pre.cluster” and “chimera.uchime” to reduce the sequencing errors. Sequence classifications were carried out with the Mothur implementation of the RDP database using the “classify.seqs” command. Sequences identified as “Mitochondria,” “Chloroplast,” “Archaea,” “Eukaryota,” or “unknown” were removed using the “remove.lineage” command. Distance matrices were generated with “dist.seqs” command and OTUs were obtained using the “cluster.split” command (method = average, processors = 2, splitmethod = classify, large = T). Removal of the singleton reads was carried out using the “split.abund” command and then, to have an equal number of reads per sample, datasets were standardized by a random sub-sampling using the “sub.sample” command. OTU tables at 97% similarity were generated following the steps for “create.database.”

Overall, the steps for soil and root ITS datasets processing were similar to those for 16S rRNA gene datasets processing. Most of the modifications were introduced owing to the absence of reference database for ITS sequence alignments. After elimination of low quality and short sequences by the “trim.seqs” command (maxambig = 0, maxhomop = 8, bdiffs = 1, pdiffs = 2, qwindowaverage = 30, qwindowsize = 50, minlength = 250), we ran “chop.seqs” and “chimera.uchime” commands to standardize sequence length (numbases = 249) and reduce the sequencing errors, respectively. Then, the “.fasta” files were clustered into OTUs at 97% identity using the CD-HIT software (Li and Godzik, 2006) and reformatted to “.list” file in order to continue Mothur processing. The remaining steps of the ITS datasets processing were the same as 16S rRNA gene datasets processing, except sequence classification was performed using the UNITE_97_v6 reference database, and the “remove.lineage” command was not used in ITS datasets processing.

### Statistical Analysis

All statistical analyses were performed in R (version 3.1.1). To compare the microbial diversity and OTU richness between soil and root datasets, Student’s *t*-tests or Wilcoxon tests (depending on the normality of the residuals) were carried out on Shannon diversity indices and the Chao richness estimator using the “Rcmdr” package. Student’s *t*-tests were performed on the fungal Chao values (normally distributed), while Wilcoxon tests were performed on the bacterial Chao and Shannon values, and on the fungal Shannon values (non-normally distributed residuals). Student’s *t*-tests or Wilcoxon tests were also performed to compare the most abundant fungal and bacterial classes relative abundances between soil and roots. To verify the efficiency of our sampling efforts and sequencing depth, rarefaction curves were drawn for each individual sample using the “rarefy” function in the “vegan” package. Depending on the normality of the residuals, the effect of contamination levels and plant species identity on Shannon diversity indices of fungi and bacteria were tested by ANOVA or Kruskal–Wallis tests using the “Rcmdr” package in R (ANOVA tests were carried out on soil fungi, root fungi and soil bacteria data, while Kruskal–Wallis tests were performed on root bacteria data.

To test the effect of contamination levels, plant species and habitats (soil, roots, and AMF spores) on the bacterial and fungal community structures, PERMANOVA analyses were performed using the “adonis” function in the “vegan” package on Bray–Curtis values obtained from the community structure matrices previously normalized by the Hellinger transformation (Legendre and Legendre, 2012). For the PERMANOVA carried out across habitats, we did two PERMANOVA tests. The first was performed to compare between the microbial (bacterial and fungal) communities present in soil and roots, while the second was performed to compare between the microbial communities present in the three habitats (soil, roots, and AMF spores). As the AMF spore-associated fungi dataset did not contain OTU matching with Glomeromycota, all the taxa matching with Glomeromycota in soil and root matrices were removed, prior to the PERMANOVA test preformed to compare the fungal communities between the three habitats. Since the microbial datasets of soil, roots and AMF spores were obtained from different sequencing pools with different sequencing depth, the matrices were first summed at the genera level and then expressed as percentages, prior to the merging of matrices, rarefaction of data and PERMANOVA analyses. The percentages were performed by calculating the report of abundance of each OTU per site on the sum of OTUs abundances per site. To test the homogeneity of dispersion of the different communities against PHP concentration and plant species identity, beta-dispersion analyses were performed on the Bray–Curtis matrices using the “betadisper” function in the “vegan” package. To reveal which fungal or bacterial taxa were affected by the contamination levels or plant species, Kruskal–Wallis tests or ANOVA were performed on the abundances at class level and on the most abundant thirty OTUs of fungi and bacteria in both soil and root datasets. Non-metric multidimensional scalings (NMDS) were performed to visualize the effects of contamination levels, plant species and biotopes on community composition. NMDS ordinations were calculated from the Bray–Curtis matrices using the “metaMDS” function from the vegan package. Krona charts were prepared using the KronaTools^2^ (Ondov et al., 2011) to compare the AMF-associated microbial communities (Iffis et al., 2016) with the soil and roots microbial communities.

## Results

### Soil Microbial Diversity versus Root Microbial Diversity

After quality filtering and standardizing the number of sequences in the different datasets, the soil 16S rRNA gene dataset allowed us to retrieve a total of 23085 reads (855 per sample) which were assigned to 4083 OTUs, while a total of 25839 reads (957 per sample) were obtained from the soil ITS dataset and were assigned to 215 OTUs. For the root 16S rRNA gene dataset, we retrieved a total of 2403 reads (89 per samples) which were assigned to 820 OTUs, while a total of 13716 reads (508 per samples) were obtained from the root ITS dataset and assigned to 188 OTUs.

**FIGURE 1:**
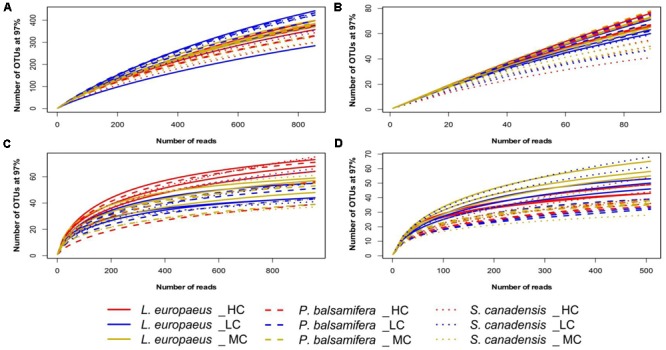
Rarefaction curves of OTUs for individual samples across the different datasets: **(A)** soil bacteria, **(B)** root bacteria, **(C)** soil fungi, **(D)** root fungi.

Rarefaction curves showed that the sampling effort for fungi was close to saturation for all samples with Good’s coverage values ranging between 0.97 and 0.99 for soil fungi, and 0.94 and 0.98 for root fungi. Contrary to fungi coverage, the sampling efforts were relatively low yielded for bacteria, in particular for the samples related to *L. europaeus* and *P. balsamifera*. The Good’s coverage values of bacteria ranged between: 0.68 and 0.81 for soil bacteria, and between 0.22 and 0.70 for root bacteria (**Figure 1** and Supplementary Table S1).

**FIGURE 2:**
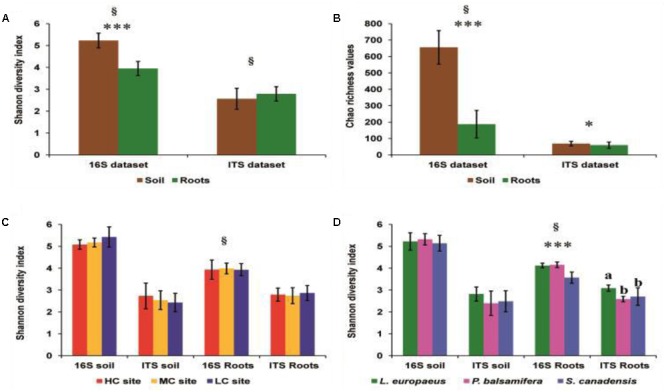
**(A)** Comparison of Shannon diversity indices of bacteria and fungi across rhizosphere soil and roots. **(B)** Comparison of Chao estimator values of bacteria and fungi across rhizosphere soil and roots. **(C)** Comparison of Shannon diversity indices of the different pyrosequencing datasets across contamination concentrations. **(D)** Comparison of Shannon diversity indices of the different pyrosequencing datasets across plant species. HC, high contamination; CM, moderate contamination, and LC, low contamination. ^∗^: significant at 5%; ^∗∗∗^: significant at 0.1%; ^§^*P-*values calculated using Wilcoxon or Kruskal–Wallis tests. Different letters over columns “a and b” indicate significant differences according to Tukey’s range tests.

The Shannon diversity indices and Chao richness estimators (**Figures 2A,B**) of bacterial OTU were significantly higher in soils than in roots (Wilcoxon test, *P* < 0.0001). For fungi, Wilcoxon test on Shannon diversity indices showed no significant difference in fungal diversity between soil and root datasets (*P =* 0.15). However, a significant difference in OTU richness was observed between the soil and root samples for fungi (Student’s *t*-test, *P* = 0.05), with Chao values higher in the soil samples (mean Chao value = 68.66) compared to root samples (mean Chao value = 59.58) (**Figures 2A,B**).

**FIGURE 3:**
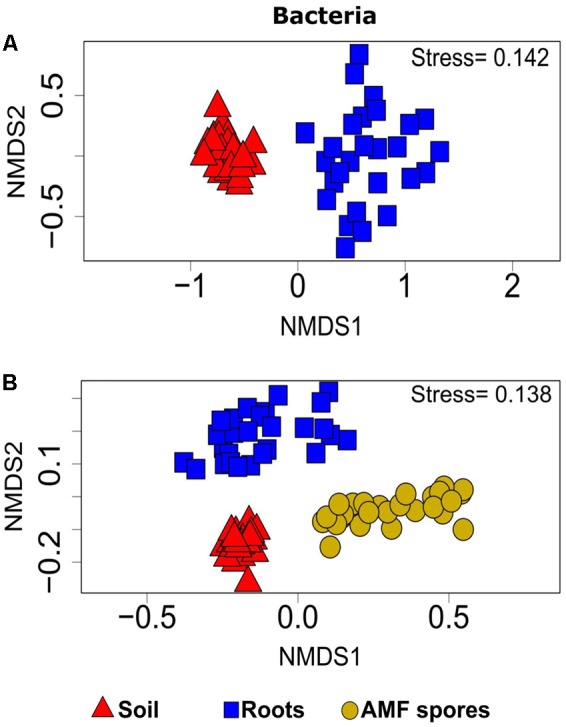
Non-metric multidimensional scaling (NMDS) showing the bacterial community compositions assignments across: **(A)** rhizosphere soil and roots (stress value = 0.14, PERMANOVA, *P* < 0.001), **(B)** rhizosphere soil, roots and AMF spores (stress value = 0.13, PERMANOVA, *P* < 0.001).

**FIGURE 4:**
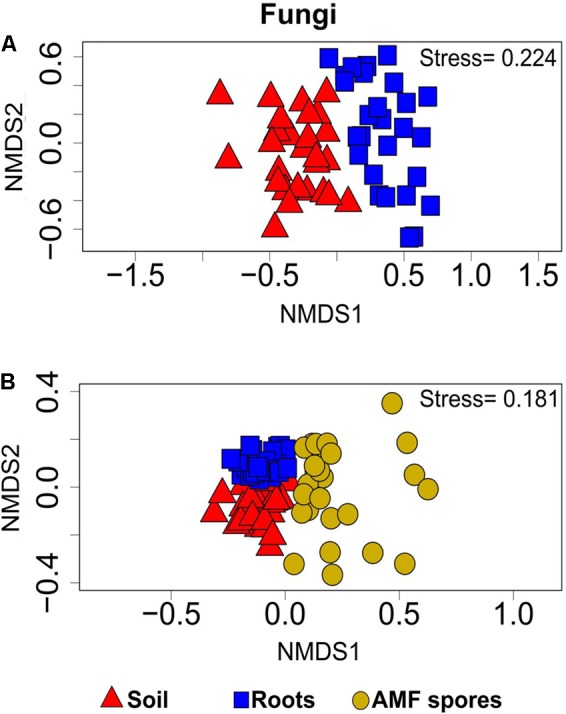
Non-metric multidimensional scaling showing the fungal community compositions assignments across: **(A)** rhizosphere soil and roots (stress value = 0.22, PERMANOVA, *P* < 0.001), **(B)** rhizosphere soil, roots and AMF spores (stress value = 0.18, PERMANOVA, *P* < 0.001).

PERMANOVA analyses showed that the bacterial and fungal community structures present in soil were significantly different from the communities found in association with plant roots (*P* < 0.001). For both bacteria and fungi, NMDS ordinations showed a clear change in community structures across the two habitats (stress value = 0.14 and 0.22, respectively) (**Figures 3A, 4A**).

BLAST searches of the bacterial 16S rRNA gene sequences showed that, at class or phylum level, the bacterial profiles were similar in soil and root datasets. The most abundant bacterial taxa identified in the two datasets belong to classes *Alphaproteobacteria Betaproteobacteria, Gammaproteobacteria*, and phyla *Actinobacteria* and *Acidobacteria*. However, except for *Alphaproteobacteria* and *Betaproteobacteria*, which were found approximately in similar proportions in soil and root datasets (36 and 33% for *Alphaproteobacteria*, and 14 and 12% for *Betaproteobacteria*), the proportions of the other most abundant bacterial groups were different between the soil and root datasets. *Acidobacteria* and *Deltaproteobacteria* had higher proportions in the soil dataset (11 and 5.25%, respectively) compared to the root dataset (4 and 3.41%, respectively) (Student’s *t*-test, *P* < 0.05) while, the percentages of *Actinobacteria* (represented mainly by *Streptomyces*) and *Gammaproteobacteria* were higher in the root dataset (19 and 14%, respectively) than in the soil dataset (6 and 9%, respectively) (Wilcoxon test, *P* < 0.0001 for *Actinobacteria* and Student’s *t*-test = 0.15 for *Gammaproteobacteria*) (Supplementary Figures S2A,B). In addition, within each bacterial group, abundances and taxonomic affiliations of the most OTUs found in the root dataset were different from those found in the soil dataset (Supplementary Figures S3A,B). For instance, in the root dataset, the OTUs related to *Alphaproteobacteria* were represented mainly by *Bradyrhizobium, Skermanella, Sphingobium*, and *Hoeflea*, while in the soil dataset, *Alphaproteobacteria* were dominated by *Sphingomonas, Skermanella*, and *Dongia*. Similarly, *Betaproteobacteria* were represented mainly by *Ideonella, Duganella*, and *Limnobacter* in the root dataset, while they were dominated by *Caenimonas, Burkholderiales*, and *Ferrovum* in the soil dataset.

For the ITS sequences, BLAST searches showed that almost all fungal taxa identified in the soil dataset were also identified in the root dataset. Most fungal taxa found in the two datasets belong to the classes *Dothideomycetes, Sordariomycetes, Agaricomycetes, Chytridiomycetes*, and *Glomeromycetes*. *Dothideomycetes, Agaricomycetes*, and *Chytridiomycetes* were found in similar proportions in the root and soil datasets. *Dothideomycetes* represented 23 and 24% of the OTUs in soil and root datasets, respectively. *Agaricomycetes* represented 10 and 7% of OTUs in the soil and root datasets, respectively. Similarly, *Chytridiomycetes* represented 5 and 4% of OTUs in the root and soil datasets, respectively. However, the proportions of *Sordariomycetes* and *Glomeromycetes* were clearly different between soil and root samples (Wilcoxon test, *P* ≤ 0.0001). *Sordariomycetes* were found as the most dominant taxa in the soil dataset, with 37% of OTUs, whereas they represented only 5% of the root dataset. *Glomeromycetes* represented 20% of the root dataset, but only 1% of the fungal sequences in the soil dataset (Supplementary Figures S2C,D). At the genera level, abundances and taxonomic affiliation of the fungal OTU found in roots were also different from those found in soil. For example, *Dothideomycetes* were found at similar proportions in both soil and roots habitats (23% in soil and 24% in roots), however, the *Dothideomycetes* genera found in soil and roots were different. *Leptosphaeria* was the most dominant genus of *Dothideomycetes* found in roots (37%), while unclassified *Pleosporales* was the most dominant *Dothideomycetes* found in soil (42%) and *Leptosphaeria* represent only 10% (Supplementary Figures S4A,B).

### PHP and Plant Species Identity Effects on Soil and Root Bacterial Diversity

ANOVA tests revealed a nearly significant effect of the contamination concentration on Shannon diversity indices of bacteria in the soil dataset (*P =* 0.077), with a slightly higher diversity in the LC site than in HC, and MC sites. On the other hand, there was clearly no effect of contamination on the bacterial diversity indices in the root dataset (**Table 1** and **Figure 2C**). However, when comparing the shifts in Shannon diversity indices by plant species, a highly significant effect of plants species identity on bacterial diversity was observed in the root dataset (*P* < 0.001), with a higher diversity of bacteria in *L. europaeus* and *P. balsamifera* than in *S. canadensis*. In contrast, there was no effect of plant species identity on the bacterial diversity in the soil dataset (**Table 1** and **Figure 2D**).

**Table 1:** *P*-values of ANOVA and PERMANOVA analyses of the effects of contamination level and plant species identity on the Shannon diversity indices and community structures of bacterial and fungal communities present in soils and roots of *Solidago canadensis, Populus balsamifera*, and *Lycopus europaeus* growing spontaneously in three petroleum hydrocarbon polluted sedimentation basins.

	Contamination level			Plant species		
	ANOVA on Shannon index	PERMANOVA on the community structure	Beta-dispersion analysis	ANOVA on Shannon index	PERMANOVA on the community structure	Beta-dispersion analysis
Soil bacteria	0.0777	**0.011**	0.25	0.544	0.428	0.49
Root bacteria	§0.7242	**0.034**	0.46	§**0.0001**	**0.001**	**0.031**
Soil fungi	0.424	**0.002**	0.72	0.151	0.37	0.10
Root fungi	0.714	**0.006**	0.51	**0.000965**	**0.002**	0.06

*The bolded values are significant at *P* < 0.05. § *P*-values calculated using Kruskal–Wallis tests. For each group, *n* = 9.*

PERMANOVA analysis revealed that the community structure of bacteria was significantly affected by the contamination level for both soil and root datasets (*P* = 0.01 and 0.034, respectively). However, a significant effect of plant species identity on the bacterial community structure was observed only on the root dataset (*P* = 0.001), while no effect of plant species was observed on bacteria in the soil dataset (*P* = 0.42) (**Table 1**).

**FIGURE 5:**
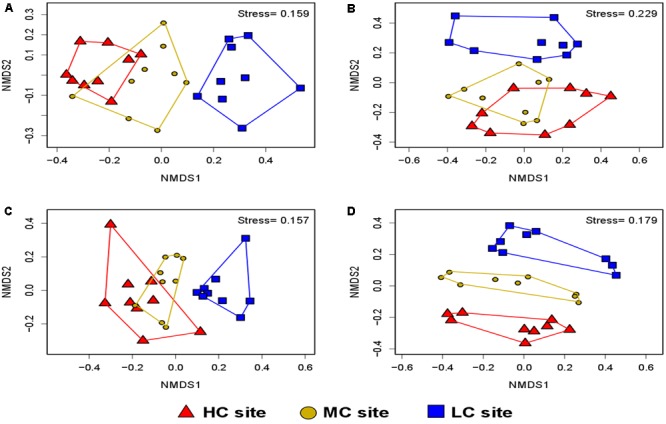
Non-metric multidimensional scaling showing the community composition assignments of: **(A)** soil bacteria (stress value = 0.15, *P* = 0.011), **(B)** root bacteria (stress value = 0.22, *P* = 0.034), **(C)** soil fungi (stress value = 0.15, *P* = 0.002), and **(D)** root fungi across contamination concentrations (stress value = 0.17, *P* = 0.006). PERMANOVA analysis showed significant effects of contamination levels on the community composition of soil bacteria, root bacteria, soil fungi and root fungi (*n* = 9).

**FIGURE 6:**
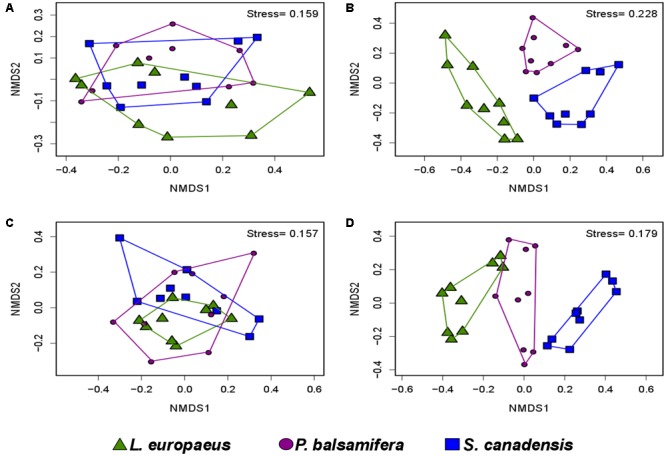
Non-metric multidimensional scaling showing the community compositions assignments of: **(A)** soil bacteria (stress value = 0.15), **(B)** root bacteria (stress value = 0.22), **(C)** soil fungi (stress value = 0.15), and **(D)** root fungi per plant species identity (stress value = 0.17). PERMANOVA analysis showed significant effects of plant species identity only on the community composition of root bacteria and root fungi (*n* = 9, *P* = 0.001 and 0.002, respectively).

The variations in diversity and community composition of bacteria were corroborated by the non-metric multidimensional scaling (NMDS) plots in both soil and root datasets. In NMDS plots performed across contamination concentrations, a clear separation of the bacterial communities was observed between the LC and HC sites in both soil and root datasets (stress value = 0.15 and 0.22, respectively) (**Figures 5A,B**). However, the NMDS plot performed across plant species showed that the bacterial communities were determined by plant species identity only in the root dataset, where bacteria from *S. canadensis* roots grouped apart those from the other plant species (stress value = 0.15 and 0.22, respectively) (**Figure 6A,B**).

Kruskal–Wallis tests performed on the soil bacterial OTUs showed clearly that the contamination level had a stronger effect on the bacterial composition than plant species (Supplementary Table S2). Among the most abundant 30 OTUs, 17 were significantly affected by the contamination concentrations, two by plant species, and two by both factors, while no effect was found on the remaining nine OTUs. Most of the OTUs affected by contamination belong to *Alphaproteobacteria* (*Sphingomonas, Skermanella, Dongia, Rhizobiales*), *Betaproteobacteria* (*Caenimonas, Burkholderiales, Ferrovum, Comamonadaceae*), *Gammaproteobacteria* (*Xanthomonadales, Thermomonas, Steroidobacter*) and *Acidobacteria* groups (Supplementary Table S2).

**FIGURE 7:**
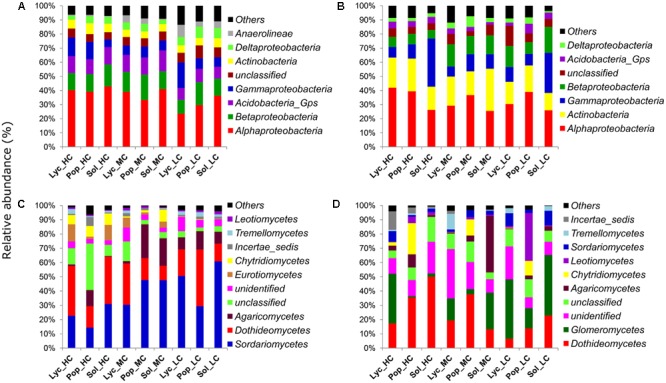
Relative abundances of major: **(A)** soil bacteria classes, **(B)** root bacteria classes, **(C)** soil fungi classes and **(D)** root fungi classes. Sol_HC, *Solidago canadensis* in the highly contaminated site; Pop_HC, *Populus balsamifera* in the highly contaminated site; Lyc_HC, *Lycopus europaeus* in the highly contaminated site; Sol_MC, *S. canadensis* in the moderately contaminated site; Pop_MC, *P. balsamifera* in the moderately contaminated site; Lyc_MC, *L. europaeus* in the moderately contaminated site; Sol_LC, *S. canadensis* in the least contaminated site; Pop_LC, *P. balsamifera* in the least contaminated site; Lyc_LC, *L. europaeus* in the least contaminated site.

At class level, the proportions of *Alphaproteobacteria* and *Acidobacteria* groups were significantly increased in the HC (40.8 and 11.6%) and MC (37.7 and 12.9%) sites than in the LC site (29.8 and 8.7%) (ANOVA, *P* = 0.012 and 0.002). *Betaproteobacteria* showed also a slightly higher proportion in the HC and MC sites (13.3 and 15%) than in the LC site (12.8%), though ANOVA test did not show a significant difference. On the other hand, the abundance of *Gammaproteobacteria* was slightly higher in the LC and HC sites (10.8 and 10.4%) than in the MC site (7.2%), though the difference was also not significant (**Figure 7A**).

Kruskal–Wallis tests performed on the root bacterial OTUs showed that the most abundant OTUs were affected by both contamination concentrations and plant species identity. Among the 30 most abundant OTUs, 10 were significantly affected by the contamination concentration, 13 by plant species and two by both contamination and plant species. Bacterial OTU related to *Alphaproteobacteria* (*Bradyrhizobium, Skermanella, Sphingobium, Hoeflea, Hyphomicrobium*, and *Altererythrobacter*), *Actinobacteria* (*Streptomyces, Actinoplanes, Streptomyces*, and *Lentzea*), *Betaproteobacteria* (*Ideonella, Duganella* and *Limnobacter*) and *Gammaproteobacteria* (*Rhizobacter, Steroidobacter*, and *Pseudomonas*), were the most affected by contamination levels or plant species (Supplementary Table S2).

When comparing the relative abundances of the root bacteria at class or phyla level across the contaminated sites, we observed that *Actinobacteria* were in higher proportions in the HC (20.4%) and MC sites (22.5%) than in the LC site (15.6%) (ANOVA, *P* = 0.041). On the other hand, *Betaproteobacteria* class was in higher abundance in the LC (13.9%) and MC sites (14.1%) than in the HC site (6.9%) (ANOVA, *P* = 0.003).

Across plant species, *Alphaproteobacteria* were clearly more abundant in *P. balsamifera* (38.3%) and *L. europaeus* roots (33.8%) than in *S. canadensis* (25.8%) samples (ANOVA, *P* < 0.001). By contrast, the abundance of *Gammaproteobacteria* was higher in *S. canadensis* (24.2%) than in *P. balsamifera* (10.1%) and *L. europaeus* (8.4%) roots (Kruskal–Wallis, *P* = 0.004). For *Betaproteobacteria*, their abundance was slightly higher in *S. canadensis* and *L. europaeus* roots (12.6% in both plant species), compared to *P. balsamifera* (9.6%), but the *P*-value was not significant (**Figure 7B**).

### PHP and Plant Species Identity Effects on Soil and Root Fungal Diversity

ANOVA tests showed that there was no effect of contamination on the Shannon diversity indices of fungi in either soil (*P* = 0.424) or root (*P* = 0.714) datasets (**Table 1** and **Figure 2C**). However, there was a highly significant effect of plant species identity on the fungal diversity in roots (*P* < 0.001). Tukey’s range test showed that the divergence in root fungal diversity has occurred in *L. europaeus*, which showed the highest diversity compared with *P. balsamifera* and *S. canadensis*. No effect of plant species identity on the fungal diversity was found in soil (**Table 1** and **Figure 2D**).

PERMANOVA analysis showed that contamination had a significant effect on the community structure of fungi in both soil and root samples (*P* = 0.002 and *P* = 0.006, respectively), while plant species identity had a significant effect in roots only (*P* = 0.002) (**Table 1**). NMDS plots showed a clear separation between the community composition of the HC and LC sites, while the community of the MC site was intermediary in both soil and root datasets (**Figures 5C,D**). NMDS plots across plant species showed differences in community structure in roots only, where a distinct grouping of the fungal communities was found between *L. europaeus* and *P. balsamifera*, with the community of *S. canadensis* being intermediate (**Figures 6C,D**).

The Kruskal–Wallis tests confirmed that soil fungi were more affected by the contamination levels than by plants species identity, while a similar amount of root fungi were affected by either contamination and plants species (Supplementary Table S2). Among the 30 most abundant soil fungal OTUs, 13 were significantly affected by contamination, four by plants species, one by both, while the 12 remaining OTUs were not affected. The soil fungal OTUs significantly affected by contamination concentration or plants species identity belong mainly to the fungal classes *Sordariomycetes* (*Emericellopsis* sp. [OTU 5], *Lasiosphaeriaceae* [OTU 13] and *Fusarium* sp. [OTU 19]), *Agaricomycetes* (*Thelephoraceae* [OTU 13]), *Dothideomycetes* (*Leptosphaeria* sp. [OTU 11] and *Pycnidiophora* sp. [OTU 27]), *Eurotiomycetes* (*Penicillium* sp. [OTU 6]), and *Chytridiomycetes* (*Spizellomyces plurigibbosus* [OTU 8]). On the other hand, among the 30 most abundant root fungal OTUs, nine were affected by contamination, 10 by plants species, eight by both, while three OTUs were not affected. Most of the root fungal OTUs significantly affected by contamination levels and/or plant species identity belong to the classes *Dothideomycetes* (*Leptosphaeria* sp. [OTU 1], *Pleosporales* sp. [OTU 15] and *Phoma herbarum* [OTU 7]), *Chytridiomycetes* (*Olpidium brassicae* [OTU 6] and *S. plurigibbosus* [OTU 2]), *Glomeromycetes* (*Claroideoglomus* [OTU 10] and *Entrophospora infrequens* [OTU 4]), *Leotiomycetes* (*Helotiales* [OTU 9]) and *Sordariomycetes* (*Fusarium sacchari* [OTU 22] and *Myrothecium* sp. [OTU 29]) and *Agaricomycetes* (*Sebacinaceae* [OTU 5]).

In soil, at class level, *Sordariomycetes* showed more abundance in the LC (46.9%) and MC sites (41.9) than in the HC site (22.6%) (Kruskal–Wallis, *P* = 0.02). Contrarily, *Eurotiomycetes* and *Chytridiomycetes* were more abundant in the HC site (8.1 and 7.3%) than in MC (4.1 and 3.7%) and LC sites (0.1 and 0.3%) (Kruskal–Wallis, *P* ≤ 0.001) (**Figure 7C**). *Dothideomycetes* were also slightly more abundant in the HC (27.7%) than MC and LC sites (18.1 and 23.7%), though the difference was not significant by Kruskal–Wallis rank test. In roots, *Dothideomycetes* and *Chytridiomycetes* were more abundant in the HC site (34.3 and 8.1%) than in MC (23.4 and 3.9%) and LC sites (14.4 and 3.6%) (Kruskal–Wallis, *P* ≤ 0.05). Contrarily, *Glomeromycetes* and *Sordariomycetes* were more abundant in the LC site (32.8 and 7.3%) compared to MC (14.9 and 3.2%) and HC sites (12.7 and 3.7%) (Kruskal–Wallis, *P* = 0.02 and 0.04). When the abundances of root fungi were compared across plant species, we observed that the proportions of OTUs belonging to different fungal classes also varied between plant species identity. *Dothideomycetes* were slightly more abundant in *P. balsamifera* (29%) and *S. canadensis* (28.7%) than in *L. europaeus* (14.4%) (Kruskal–Wallis, *P* = 0.09), while *Glomeromycetes* were more abundant in *L. europaeus* (30.7%) and *S. canadensis* (23.5) than in *P. balsamifera* (6.3%) (Kruskal–Wallis, *P* = 0.004). *Agaricomycetes* were in higher proportions in *S. canadensis* (14.7%) compared to *L. europaeus* (1.6%) and *P. balsamifera* (5.3%) (Kruskal–Wallis, *P* = 0.018). *Chytridiomycetes* and *Leotiomycetes* were more abundant in *P. balsamifera* (14.5 and 13.2%, respectively) than in *L. europaeus* (1 and 0.6%, respectively) and *S. canadensis* (0.04 and 1.2%, respectively) (Kruskal–Wallis, *P* < 0.001) (**Figure 7D**).

### Soil and Root Microbial Diversity versus AMF Spore-Associated Microbial Diversity

The comparison of the soil and root microbial communities with those identified in association with AMF spores harvested from the same plant rhizospheres (Iffis et al., 2016) revealed that the community structure of AMF-associated microorganisms significantly differed from the communities identified in the rhizospheric soil and plant roots (PERMANOVA, *P* < 0.001 for both bacteria and fungi). The NMDS plots showed a distinct grouping of soil and root microbial communities compared with AMF spore-associated microbial communities (**Figures 3B, 4B**). We previously found that *Gammaproteobacteria* and *Betaproteobacteria* were the most dominant classes associated within AMF spores (their abundances were 49 and 23%, respectively), while the most dominant fungi belonged to the unclassified fungi (55%), *Pezizomycetes* (13%) and *Dothideomycetes* (13%) (Iffis et al., 2016). In the present study, *Alphaproteobacteria* was the most dominant bacterial class in both soil and root datasets (36% in soil and 33% in roots), while *Sordariomycetes* was the most dominant fungal group in soil (37%) and *Dothideomycetes* was the most dominant in roots (24%). Here, *Gammaproteobacteria* represented only 9% of OTUs in soil and 14% in roots, while *Pezizomycetes* formed only 1.3% of OTUs in roots and 0.8% in soil (Supplementary Figure S5). The pezizomycete OTU was represented by *Sphaerosporella brunnea*, an ectomycorrhizal species that we previously found abundant in willow roots grown in the same contaminated site (Bell et al., 2014). However, here we did not find any significant effect of plant species on this fungal OTU (Supplementary Table S2). Differences in community structures between the AMF spore-associated microbiomes and the rhizospheric and root microbiome were also found at the genus level (Iffis et al., 2016). Indeed, *Alphaproteobacteria* OTU were represented mainly by the genera *Sphingomonas* in soils and *Bradyrhizobium* in roots, while this group was represented mainly by the genus *Caulobacter* in the AMF spore-associated microbiome (Iffis et al., 2016). Similarly, *Betaproteobacteria* OTU were represented mainly by the genera *Duganella* and *Janthinobacterium* in the spore microbiome, while they were formed mainly by unclassified *Betaproteobacteria* and *Caenimonas* in soil, and by *Duganella* in roots (Supplementary Figure S3). For fungi, *Septoria* was the most representative genus of *Dothideomycetes* associated with the AMF spores, while in soil and roots, the *Dothideomycetes* were represented mainly by *Pleosporales* (Supplementary Figure S4).

## Discussion

In rhizospheric soil, plant roots, bacteria and fungi form tripartite associations ranging from beneficial to harmful interactions based on exchange of complex signaling dialogs and nutrient compounds by which each partner influences the other to avoid the different biotic and/or abiotic stresses able to disrupt their life cycle. Therefore, the microbial communities living in soil or in association with roots are intimately linked to the different exudates released in the rhizosphere (root and microbial exudates), to soil composition and to climatic conditions. In this study, we assessed the variation in bacterial and fungal diversity across PHP concentrations, plant species identity and habitats (soil versus roots). Furthermore, taking advantage of the fact that soil and root samples used in this study were the same as those used previously in Iffis et al. (2016) study, we compared the bacterial and fungal diversity found in the current study to that found associated with the AMF spores in the Iffis et al. (2016) study to test the hypothesis that microbial communities living in association with AMF spores are selected by the AMF and not only randomly recruited from those found in the rhizosphere and roots of the host plants.

In our study, rarefaction curved of bacterial diversity were not saturated because of the limited number of sequences per sample, thus only dominating bacterial taxa are discussed. The comparison of microbial communities across PHP concentrations revealed that *Alphaproteobacteria* were favored in the high contaminated HC site, both in soil and in roots. *Actinobacteria* were also among the most dominant groups in the plant roots of the HC site. The high abundance of *Alphaproteobacteria* and *Actinobacteria* in the HC site may be related to their PHP tolerance and/or their ability to degrade PHP compounds. Several studies carried out on the microbial communities in PHP contaminated sites showed that *Alphaproteobacteria* and *Actinobacteria* were often found in higher abundances in soils containing high amounts of organic contaminants (Greer et al., 2010; Bell et al., 2014; Yergeau et al., 2014; Pagé et al., 2015). Furthermore, *Sphingomonas* (the most dominant *Alphaproteobacteria* in the soil dataset), *Bradyrhizobium* (the most dominant *Alphaproteobacteria* in the root dataset), and *Streptomyces* (the most dominant *Actinobacteria* in soil and roots) were shown to degrade a range of recalcitrant PAH compounds, such as phenanthrene, pyrene, and naphthalene (Rentz et al., 2008; Qu and Spain, 2011; Balachandran et al., 2012; Bourguignon et al., 2014). The presence of *Gammaproteobacteria* in similar abundances in the LC and HC sites, both in soil and root datasets, may be related to the large spectrum of activities of the species belonging to this class. For example, in PHP contaminated soils, *Pseudomonas* (the most dominant genus of *Gammaproteobacteria* in roots) was shown to degrade a range of PAH compounds such as phenanthrene, alkane, and naphthalene (Ma et al., 2006; Ní Chadhain and Zylstra, 2010; Sun et al., 2014). On the other hand, in agricultural soils, *Pseudomonas* taxa are known as potential plant growth-promoting bacteria able to establish a symbiotic association with plant roots and to play an important role in plant growth, nitrogen fixation and phosphate solubilization (Rodrìguez and Fraga, 1999; Desnoues et al., 2003; Sharma et al., 2013). In the case of fungi, *Dothideomycetes* and *Chytridiomycetes* were the fungal classes found in higher abundance in the HC site. To our knowledge, PHP tolerance or biodegradation abilities of *Chytridiomycetes* was never studied. Surprisingly, except the study of Iffis et al. (2016), none of the published studies carried out nearby the basins of our study have found *Chytridiomycetes*, either in rhizospheric soils and sediments or in association with plant roots (Bell et al., 2014; Stefani et al., 2015; Bourdel et al., 2016). However, these studies found that *Dothideomycetes* were among the most dominant fungal classes in the PHP contaminated sites. Furthermore, several studies reported that some species belonging to *Dothideomycetes* are able to tolerate or break down a range PHP compounds (Junghanns et al., 2008; Ferrari et al., 2011; Harms et al., 2011; Stefani et al., 2015). For example, *Alternaria* and *Cladosporium*, which were detected here both in soil and root datasets, have been shown to degrade crude oil and a variety of its derivative products such as phenanthrene, benzo[a]pyrene, fluoranthene, and anthracene (Giraud et al., 2001; Potin et al., 2004; Mohsenzadeh et al., 2012; Ameen et al., 2016).

Our results also showed that the OTU richness of bacteria and fungi were significantly decreased in root samples in comparison to the soil samples. Generally, the microbial diversity was shown to increase in rhizospheric soils compared to the different plant compartments (roots, stem, or leaves) (Xu et al., 2012; Turner et al., 2013a; Edwards et al., 2015). The increase of microbial richness in soils compared to roots may be related to the difference in environmental conditions and nutrient bioavailability in the two ecological niches (soil versus roots). Indeed, plant roots have a selective effect on both rhizospheric and endophytic microorganisms, however, the selective effect is much higher in the endosphere (inside roots) owing to the complexity and specificity of plant–microbe interactions and plant immune system responses (Bais et al., 2006; Hardoim et al., 2008; Oldroyd, 2013). Generally, before root colonization, plants and microorganisms engage in a complex chemical dialog, and only the bacteria or fungi recognizing the signaling pathways are allowed to penetrate and colonize plant roots (Bertin et al., 2003; Bais et al., 2006; Oldroyd, 2013). Furthermore, once inside roots, the endophytes are subjected to stress caused by the new conditions and consequently, only the microbes able to adapt to the intraradical conditions can proliferate inside root compartments (Jones et al., 2007; Parniske, 2008; Gaiero et al., 2013; Brader et al., 2014). For example, plant root infection by nitrogen fixing bacteria (e.g., *Bradyrhizobium*) and arbuscular mycorrhizal fungi, which are found in higher proportion in root samples than in soil samples, is achieved through an exchange of complex chemical signaling between the plant roots and microbes (Bonfante and Anca, 2009; Philippot et al., 2013). In the nitrogen-fixing bacterial symbiosis, plant roots release in the rhizosphere specific signaling compounds, composed mainly of flavonoids, which stimulate these bacteria to produce a series of lipochitooligosaccharide compounds (nodulation factors) that are required to activate the rest of the symbiosis signaling pathway (Fisher and Long, 1992; Jones et al., 2007). An analog strategy to nitrogen-fixing bacterial infection was also described between plant roots and AMF. The signaling begins with root exudation of strigolactones in the rhizosphere (Parniske, 2008). Perception of strigolactones by AMF stimulate the spores to answer by releasing other signaling compounds, so called “Myc factors,” which trigger the symbiosis pathway (Parniske, 2008; Maillet et al., 2011; Oldroyd, 2013). Unlike the conditions faced by microorganisms living in the roots, in the rhizosphere, the soil surrounding the roots is rich in nutrients. A large range of soil organic matter, as well as root exudates composed mainly of carbohydrates, amino acids and organic acids are present at the soil-root interface and stimulate the proliferation of the rhizosphere-living fungi and bacteria (Bertin et al., 2003; Somers et al., 2004; Bais et al., 2006; Philippot et al., 2013; Quiza et al., 2015).

The comparison of microbial communities between soil and roots showed that the proportions of OTUs belonging to some groups of fungi (e.g., *Sordariomycetes* and *Glomeromycetes*) and bacteria (e.g., *Gammaproteobacteria, Actinobacteria*, and *Acidobacteria*) were different between rhizospheric soils and plant roots. In addition, at the genus rank, we found that the community structures of fungal and bacterial genera identified in rhizospheric soils were different from those identified in plant roots. Even if the root microbiome was considered as a community derived from the rhizospheric soil (Compant et al., 2010; Gaiero et al., 2013; Turner et al., 2013a), several studies demonstrated that the microbial community composition (fungi and bacteria) in rhizospheric and bulk soils are different from those of plant roots (Smalla et al., 2001; Xu et al., 2012; Shakya et al., 2013; Edwards et al., 2015; Quiza et al., 2015). Usually, the same phyla or classes of microorganisms were found in soils and roots, but their abundances varied between the two habitats. Moreover, differences in the taxonomic affiliations were often reported when the comparisons were carried out at genus or species rank (Gottel et al., 2011; Turner et al., 2013a,b; Edwards et al., 2015). In our study, most of the bacteria and fungi identified in high proportions in roots were already known to be endophytic, mycorrhizal or obligatory biotrophic microorganisms, establishing a symbiotic or pathogenic associations with plants. For example, *Bradyrhizobium* and *Pseudomonas* (the most dominant *Alphaproteobacteria* and *Gammaproteobacteria* found in the root dataset) are plant growth-promoting bacteria able to establish endosymbiotic associations with the roots of several plant species (Fisher and Long, 1992; Kneip et al., 2007; Sharma et al., 2013). Similarly, *Glomeromycetes* are known as obligate biotrophic fungi that require a host plants for their growth and reproduction (Simon et al., 1993; St-Arnaud and Vujanovic, 2007; Smith and Read, 2008).

While there is a growing need in the world to decontaminate polluted soils, microbe-assisted phytoremdiation can be an alternative biotechnology for remediation and revegetation of contaminated soils. The outcome of our results could improve photoremediation technology, particularly in highly contaminated sites by organic pollutants, by combining endophytic and mycorrhizal inoculants with appropriate plants.

The shifts in the community structures of AMF-associated bacteria and fungi across soils and roots observed in this study support the hypothesis that AMF select the microbial communities living in association with their spores and mycelia. As with plant roots, AMF may release carbon resources and other signaling molecules that make the surface of spores and mycelia favorable and then selective for the growth of specific microorganisms, as proposed previously (Roesti et al., 2005; Bharadwaj et al., 2011; Lecomte et al., 2011; Agnolucci et al., 2015; Iffis et al., 2016). For example, Bharadwaj et al. (2011) conducted *in vitro* cultures of 10 AMF-associated bacteria isolates and observed that the growth rates of the isolates were significantly increased by the addition to the culture medium of a broth medium in which AMF have been already cultured (they considered the broth medium as being rich in AMF exudates). In another study, Roesti et al. (2005) performed a cross-inoculation experiment with two AMF species (*Glomus geosporum* and *G. constrictum*) and two host plant species (*Plantago lanceolata* and *Hieracium pilosella*), and observed the AMF spore-associated bacterial communities were more determined by AMF-identity than plant species identity. However, little is known about AMF exudates composition and their effects on soil microorganisms. Therefore, further investigations on this topic will be required to fully understand the mechanisms by which AMF spores recruit their associated microorganisms.

## Conclusion

The high throughput amplicon sequencing approach used in our study allowed us to characterize the variations in bacterial and fungal communities in soils and roots across petroleum hydrocarbon concentrations and plant species. Overall, we found that bacterial and fungal communities associated to plant roots varied significantly across both PHP concentrations and plant species identity, while they were affected only by PHP concentrations in soil. Our results also showed that the bacterial and fungal OTU richness and community structures differed significantly between soil and roots. Furthermore, comparisons between the AMF spore-associated microbiome described previously in Iffis et al. (2016) and the results of the present study showed that the microbial communities living in association with AMF spores significantly differed from those found in the surrounding soil and roots.

## Author Contributions

BI: Performed the experiments, done bioinformatics and statistics and wrote the paper. MS-A: Helped to design the experiments and wrote the paper. MH: Supervised, conceived, designed the experiments and wrote the paper.

## Conflict of Interest Statement

The authors declare that the research was conducted in the absence of any commercial or financial relationships that could be construed as a potential conflict of interest.

## Footnotes

1 http://www.mothur.org/

2 https://github.com
