# Supplementary material for: Petroleum Contamination and Plant Identity Influence Soil and Root Microbial Communities While AMF Spores Retrieved from the Same Plants Possess Markedly Different Communities
Source: Front Plant Sci. 2017 Aug 8;8:1381. doi: 10.3389/fpls.2017.01381 (PMC5550799; doi:10.3389/fpls.2017.01381)
Supplement: Supplementary file 1 [file Presentation_1.PDF]

Supporting Information

Table S1. Observed richness, Chao1 estimator values and Good's coverage values for each individual sample across the different datasets. LY: *Lycopus europaeus*, PO: *Populus balsamifera*, SO: *Solidago canadensis*

	Soil bacteria			Root bacteria			Soil fungi			Root fungi		
**Samples**	**sobs**	**chao**	**coverage**	**sobs**	**chao**	**coverage**	**sobs**	**chao**	**coverage**	**sobs**	**chao**	**coverage**
**LY1-HC**	357	648.20	0.749	67	161.23	0.438	73	90.00	0.981	43	48.14	0.982
**LY1-LC**	373	668.66	0.745	63	149.25	0.483	44	59.00	0.990	53	61.25	0.976
**LY1-MC**	370	669.16	0.740	65	137.07	0.472	59	85.00	0.986	65	76.40	0.963
**LY2-HC**	378	688.67	0.727	66	193.50	0.427	68	77.07	0.982	49	70.00	0.970
**LY2-LC**	442	770.81	0.688	71	204.00	0.360	44	53.00	0.990	46	53.09	0.974
**LY2-MC**	399	639.32	0.739	72	227.55	0.337	57	67.50	0.984	55	74.13	0.965
**LY3-HC**	398	854.79	0.691	76	284.00	0.270	64	75.77	0.981	43	56.20	0.976
**LY3-LC**	284	577.45	0.784	60	121.50	0.528	55	59.00	0.991	50	61.67	0.970
**LY3-MC**	385	736.65	0.713	73	220.50	0.326	48	54.60	0.987	58	71.60	0.967
**PO1-HC**	328	555.77	0.779	74	318.13	0.292	71	84.60	0.982	36	51.17	0.972
**PO1-LC**	433	746.75	0.706	75	299.00	0.281	43	51.25	0.987	39	42.11	0.984
**PO1-MC**	389	747.57	0.716	64	260.86	0.404	39	47.25	0.987	35	38.00	0.988
**PO2-HC**	330	589.21	0.777	77	361.75	0.236	39	59.00	0.983	38	68.33	0.972
**PO2-LC**	423	750.55	0.712	62	129.57	0.506	51	59.67	0.986	32	71.00	0.974
**PO2-MC**	378	795.24	0.709	67	279.14	0.382	53	66.20	0.987	36	40.00	0.984
**PO3-HC**	371	635.24	0.742	72	209.75	0.348	56	83.14	0.979	34	41.50	0.980
**PO3-LC**	397	673.72	0.730	68	140.06	0.438	55	68.13	0.984	33	39.43	0.980
**PO3-MC**	376	693.29	0.731	78	338.67	0.225	55	67.36	0.982	38	41.11	0.984
**SO1-HC**	299	566.05	0.788	55	185.00	0.551	74	92.75	0.974	39	44.08	0.976

**SO1-LC**	353	588.44	0.770	59	120.50	0.528	55	70.55	0.980	61	77.15	0.959
**SO1-MC**	301	479.89	0.812	54	103.58	0.607	61	66.00	0.989	49	73.43	0.963
**SO2-HC**	325	502.00	0.792	41	70.55	0.708	75	93.47	0.972	44	63.43	0.967
**SO2-LC**	422	799.35	0.691	47	81.36	0.685	64	81.00	0.981	50	63.13	0.970
**SO2-MC**	323	472.65	0.813	48	82.36	0.685	37	50.75	0.989	37	42.14	0.982
**SO3-HC**	343	558.18	0.773	50	142.63	0.562	66	83.50	0.978	54	112.00	0.943
**SO3-LC**	430	728.36	0.705	47	122.43	0.629	41	56.00	0.990	68	97.25	0.947
**SO3-MC**	333	576.79	0.768	50	112.33	0.618	43	52.43	0.987	28	31.00	0.986

Table S2. *p* values of kruskal–wallis test showing the effects of contamination level and plant species identity on the OTU abundances of the: (a) most abundant thirty OTU of the 16S soil bacteria, (b) most abundant thirty OTU of the 16S root bacteria (c) most abundant thirty OTU of the ITS soil fungi, (d) most abundant thirty OTU of the ITS root fungi.

**A- Kruskal-Wallis test on the soil bacteria**				
	**OTUs affiliation**	**Class level**	**contamination level**	**plant species**
**OTU 1 (C)**	*Sphingomonas*	*Alphaproteobacteria*	0.0033	0.7989
**OTU 2 (C)**	*Unclassified Betaproteobacteria*	*Betaproteobacteria*	0.0003	0.9581
**OTU 3 (P)**	*Unclassified Rhizobiales*	*Alphaproteobacteria*	0.4508	0.0859
**OTU 4**	*Sphingomonas*	*Alphaproteobacteria*	0.3299	0.7332
**OTU 5 (C)**	*Unclassified Gammaproteobacteria*	*Gammaproteobacteria*	0.0399	0.614
**OTU 6 (C)**	*Skermanella*	*Alphaproteobacteria*	0.0138	0.7258
**OTU 7**	*Bradyrhizobium*	*Alphaproteobacteria*	0.4850	0.4854
**OTU 8 (C)**	*Caenimonas*	*Betaproteobacteria*	0.0678	0.5422
**OTU 9**	*Acinetobacter*	*Gammaproteobacteria*	0.2001	0.2091
**OTU 10**	*Bellilinea*	*Anaerolineae*	0.1566	0.1566
**OTU 11 (C.P)**	*Dongia*	*Alphaproteobacteria*	0.0582	0.0723
**OTU 12 (C)**	*Unclassified Acidobacteria Gp4*	*Acidobacteria_Gp4*	0.0002	0.8235

**OTU 13 (C)**	*Dongia*	*Alphaproteobacteria*	0.0567	0.4271
**OTU 14**	*Unclassified Deltaproteobacteria*	*Deltaproteobacteria*	0.8115	0.5443
**OTU 15 (C)**	*Steroidobacter*	*Gammaproteobacteria*	0.0051	0.9505
**OTU 16**	*Acinetobacter*	*Gammaproteobacteria*	0.3679	0.3678
**OTU 17 (C)**	*Unclassified Acidobacteria Gp4*	*Acidobacteria_Gp4*	0.0015	0.9458
**OTU 18 (C)**	*Unclassified Burkholderiales*	*Betaproteobacteria*	0.0555	0.6418
**OTU 19 (C)**	*Thermomonas*	*Gammaproteobacteria*	0.0067	0.4271
**OTU 20 (C)**	*Ferrovum*	*Betaproteobacteria*	0.0011	0.2729
**OTU 21 (C)**	*Unclassified Comamonadaceae*	*Betaproteobacteria*	0.0901	0.2268
**OTU 22 (C)**	*Unclassified Xanthomonadales*	*Gammaproteobacteria*	0.0273	0.7325
**OTU 23 (C)**	*Unclassified Burkholderiales*	*Betaproteobacteria*	0.0033	0.3724
**OTU 24 (P)**	*Unclassified Burkholderiales*	*Betaproteobacteria*	0.2148	0.0579
**OTU 25**	*Dongia*	*Alphaproteobacteria*	0.7484	0.3193
**OTU 26**	*Terrimonas*	*Sphingobacteriia*	0.5309	0.6835
**OTU 27 (C)**	*Unclassified Rhizobiales*	*Alphaproteobacteria*	0.0101	0.5183
**OTU 28**	*Skermanella*	*Alphaproteobacteria*	0.4622	0.3296
**OTU 29 (C.P)**	*Unclassified Ohtaekwangia*	*Bacteroidetes*	0.0264	0.0467
**OTU 30 (C)**	*Dongia*	*Alphaproteobacteria*	0.0135	0.5054
**B- Kruskal-Wallis test on the root bacteria**				
**OTU 1(P)**	*Pseudomonas*	*Gammaproteobacteria*	0.1154	0.0113
**OTU 2 (C)**	*Bradyrhizobium*	*Alphaproteobacteria*	0.0846	0.2454
**OTU 3 (P)**	*Pseudomonas*	*Gammaproteobacteria*	0.1115	0.0036
**OTU 4 (C)**	*Streptomyces*	*Actinobacteria*	0.0159	0.6044
**OTU 5 (C)**	*Steroidobacter*	*Gammaproteobacteria*	0.0049	0.8035
**OTU 6 (C.P)**	*Streptomyces*	*Actinobacteria*	0.0263	0.0602
**OTU 7 (P)**	*Actinoplanes*	*Actinobacteria*	0.2762	0.0581
**OTU 8 (C.P)**	*Sphingomonas*	*Alphaproteobacteria*	0.0737	0.0470
**OTU 9 (P)**	*Duganella*	*Betaproteobacteria*	0.2687	0.0005
**OTU 10 (C)**	*Rhizobacter*	*Gammaproteobacteria*	0.0416	0.2374
**OTU 11 (P)**	*Lentzea*	*Actinobacteria*	0.7349	0.0821
**OTU 12**	*Unclassified Burkholderiales*	*Betaproteobacteria*	0.1818	0.1390
**OTU 13 (P)**	*Unclassified Proteobacteria*	*Unclassified Proteobacteria*	0.4253	0.0018
**OTU 14**	*Leifsonia*	*Actinobacteria*	0.4210	0.9354
**OTU 15 (P)**	*Hyphomicrobium*	*Alphaproteobacteria*	0.6685	0.0535
**OTU 16 (C)**	*Skermanella*	*Alphaproteobacteria*	0.0939	0.2796
**OTU 17 (P)**	*Hyphomicrobium*	*Alphaproteobacteria*	0.8675	0.0667
**OTU 18 (P)**	*Steroidobacter*	*Gammaproteobacteria*	0.3218	0.0021
**OTU 19 (C)**	*Sphingobium*	*Alphaproteobacteria*	0.0932	0.3735
**OTU 20 (P)**	*Limnobacter*	*Betaproteobacteria*	0.4367	0.0097
**OTU 21 (P)**	*Pseudomonas*	*Gammaproteobacteria*	0.1985	0.0121

**OTU 22 (C)**	*Actinoplanes*	*Actinobacteria*	0.0540	0.4537
**OTU 23 (P)**	*Ideonella*	*Betaproteobacteria*	0.1494	0.0113
**OTU 24 (P)**	*Altererythrobacter*	*Alphaproteobacteria*	0.2924	0.0012
**OTU 25**	*Unclassified Gammaproteobacteria*	*Gammaproteobacteria*	0.9830	0.2018
**OTU 26 (C)**	*Hoeflea*	*Alphaproteobacteria*	0.0906	0.1533
**OTU 27 (C)**	*Actinoplanes*	*Actinobacteria*	0.0405	0.1917
**OTU 28 (C)**	*Ideonella*	*Betaproteobacteria*	0.0637	0.8015
**OTU 29**	*Acidobacteria Gp6*	*Acidobacteria_Gp6*	0.2433	0.1528
**OTU 30**	*Dongia*	*Alphaproteobacteria*	0.2673	0.2673
**C- Kruskal-Wallis test on the soil fungi**				
**OTU 1**	*Fusarium sacchari*	*Sordariomycetes*	0.1139	0.4173
**OTU 2 (C.P)**	*Unclassified Pleosporales*	*Dothideomycetes*	0.0912	0.0126
**OTU 3 (P)**	*Unclassified Thelephoraceae*	*Agaricomycetes*	0.1226	0.0160
**OTU 4**	*Unclassified Sordariomycetes*	*Sordariomycetes*	0.1315	0.7498
**OTU 5 (C)**	*Emericellopsis sp*	*Sordariomycetes*	0.0004	0.8375
**OTU 6 (C)**	*Penicillium sp*	*Eurotiomycetes*	0.0016	0.5295
**OTU 7**	*Cladosporium sp*	*Dothideomycetes*	0.4228	0.5873
**OTU 8 (C)**	*Spizellomyces plurigibbosus*	*Chytridiomycetes*	0.0002	0.9721
**OTU 9 (C)**	*Unclassified fungi*	*Unclassified fungi*	0.0090	0.5157
**OTU 10**	*Unclassified Sordariales*	*Sordariomycetes*	0.2995	0.8009
**OTU 11 (C)**	*Leptosphaeria sp*	*Dothideomycetes*	0.0546	0.1132
**OTU 12 (C)**	*endophytic ascomycete sp*	*Unidentified ascomycete*	0.0068	0.2526
**OTU 13 (C)**	*Unclassified Lasiosphaeriaceae*	*Sordariomycetes*	0.0003	0.9631
**OTU 14 (P)**	*Pleosporaceae sp*	*Dothideomycetes*	0.1683	0.0209
**OTU 15 (C)**	*Acremonium sp*	*Sordariomycetes*	0.0031	0.9489
**OTU 16**	*fungal sp QLF106*	*Unidentified fungi*	0.6794	0.1530
**OTU 17**	*Alternaria sp*	*Dothideomycetes*	0.7618	0.3890
**OTU 18 (P)**	*Unclassified Basidiomycota*	*Unclassified Basidiomycota*	0.5688	0.0034
**OTU 19 (C)**	*Fusarium sp*	*Sordariomycetes*	0.0545	0.6308
**OTU 20**	*Cadophora luteo olivacea*	*Leotiomycetes*	0.4874	0.6427
**OTU 21 (C)**	*Phoma herbarum*	*Dothideomycetes*	0.0847	0.6056
**OTU 22**	*Mortierella alpina*	*Incertae_sedis*	0.1377	0.3057
**OTU 23**	*Chalara sp*	*Incertae_sedis*	0.5635	0.1922
**OTU 24**	*Uncultured Ganoderma*	*Agaricomycetes*	0.5943	0.7733
**OTU 25 (C)**	*Sphaerosporella brunnea*	*Pezizomycetes*	0.0113	0.1154
**OTU 26**	*Cladosporium cladosporioides*	*Dothideomycetes*	0.1234	0.5300
**OTU 27 (C)**	*Pycnidiophora sp*	*Dothideomycetes*	0.0005	0.6691
**OTU 28 (P)**	*Dioszegia changbaiensis*	*Tremellomycetes*	0.7342	0.0885
**OTU 29 (C)**	*Unclassified fungi*	*Unclassified fungi*	0.0008	0.5646

**OTU 30**	*Unclassified fungi*	*Unclassified fungi*	0.3812	0.3257
		**D- Kruskal-Wallis test on the root fungi**		
**OTU 1 (P)**	*Leptosphaeria sp*	*Dothideomycetes*	0.1705	0.0006
**OTU 2**	*Unclassified Ascomycota*	*Unclassified Ascomycota*	0.9606	0.8872
**OTU 3 (P)**	*fungal sp QLF106*	*Unidentified fungi*	0.7043	0.0003
**OTU 4 (C)**	*Entrophospora infrequens*	*Glomeromycetes*	<0.0001	0.8253
**OTU 5 (C.P)**	*Unclassified Sebacinaceae*	*Agaricomycetes*	0.0099	0.0165
**OTU 6 (P)**	*Olpidium brassicae*	*Chytridiomycetes*	0.2788	<0.0001
**OTU 7 (C.P)**	*Phoma herbarum*	*Dothideomycetes*	0.0014	0.0224
**OTU 8 (C)**	*Unclassified Pleosporales*	*Dothideomycetes*	0.0405	0.6149
**OTU 9 (C)**	*Unclassified Helotiales*	*Leotiomycetes*	0.0003	0.4083
**OTU 10 (C.P)**	*Uncultured Claroideoglomus*	*Glomeromycetes*	0.0105	0.0272
**OTU 11 (P)**	*Uncultured Glomus*	*Glomeromycetes*	0.3518	0.0007
**OTU 12 (C)**	*fungal sp SUN1*	*Unidentified fungi*	0.0073	0.1138
**OTU 13 (C)**	*Unidentified Ascomycota*	*Unidentified Ascomycota*	0.0002	0.1600
**OTU 14 (C.P)**	*Chalara sp*	*Incertae_sedis*	0.0007	0.0730
**OTU 15 (C.P)**	*Pleosporales sp*	*Dothideomycetes*	0.0393	0.0393
**OTU 16 (P)**	*Rhizophagus irregularis*	*Glomeromycetes*	0.8907	0.0001
**OTU 17 (P)**	*Unclassified Glomeraceae*	*Glomeromycetes*	0.1677	0.0032
**OTU 18 (C.P)**	*Unclassified Glomeraceae*	*Glomeromycetes*	0.0584	0.0565
**OTU 19 (P)**	*Unclassified Thelephoraceae*	*Agaricomycetes*	0.8501	0.0012
**OTU 20 (P)**	*Cadophora luteo olivacea*	*Leotiomycetes*	0.2541	0.0006
**OTU 21**	*Alternaria sp*	*Dothideomycetes*	0.9722	0.2991
**OTU 22 (C)**	*Fusarium sacchari*	*Sordariomycetes*	0.0674	0.8795
**OTU 23 (C)**	*Pleosporales sp*	*Dothideomycetes*	0.0004	0.8035
**OTU 24**	*Glomus sp*	*Glomeromycetes*	0.1217	0.1217
**OTU 25 (C.P)**	*Uncultured Tetracladium*	*unidentified*	0.0036	0.0266
**OTU 26 (C)**	*Pulvinula constellatio*	*Pezizomycetes*	0.0019	0.1114
**OTU 27 (P)**	*Dioszegia changbaiensis*	*Tremellomycetes*	0.7417	<0.0001
**OTU 28 (C.P)**	*Spizellomyces plurigibbosus*	*Chytridiomycetes*	0.0453	0.0224
**OTU 29 (C)**	*Myrothecium sp*	*Sordariomycetes*	0.0020	0.2011
**OTU 30 (P)**	*Podospora communis*	*Sordariomycetes*	0.2025	0.0654

(C): significant effect across contamination level, (P): significant effect across plant species, (C.P): significant effect across contamination level and plant species

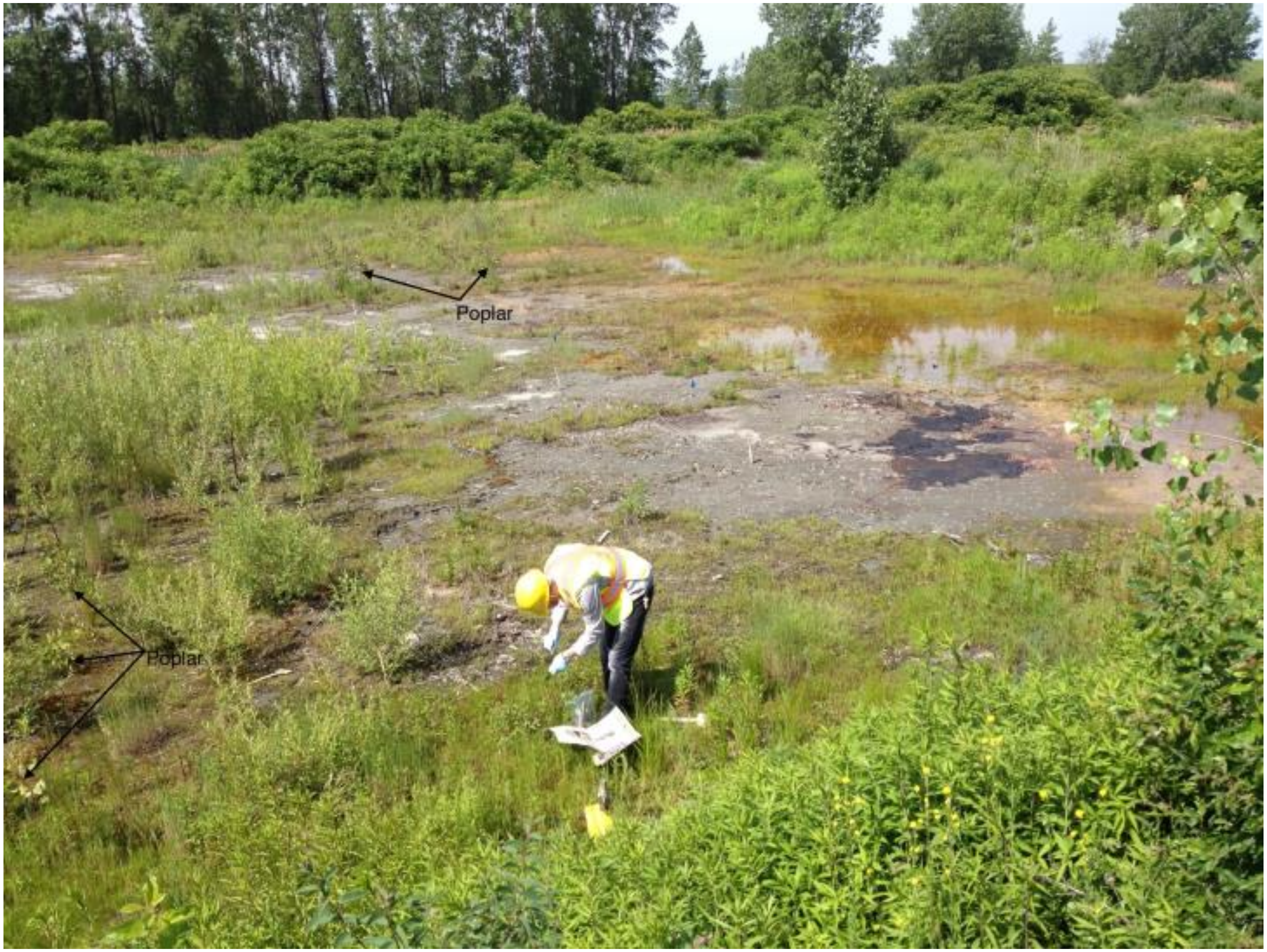

Figure S1. Illustration of highly contaminated basin where three plant species *Populus balsamifera*, *Solidago canadensis* and *Lycopus europaeus* were sampled. Poplar (*Populus balsamifera)* trees were between 0.5 and 1 m height and they are indicated by arrows.

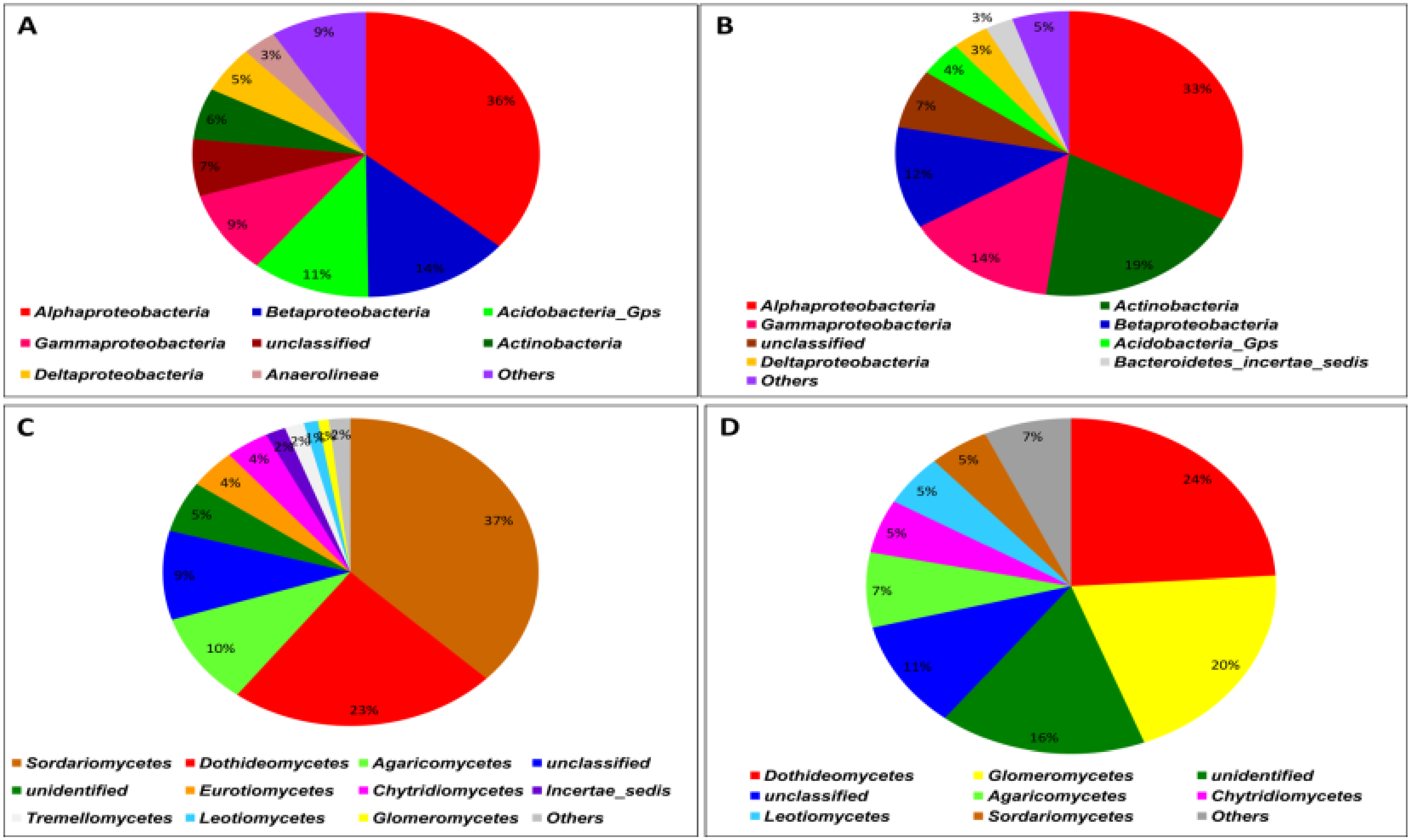

Figure S2. Proportion of the different: (A) soil bacteria classes, (B) root bacteria classes, (C) soil fungi classes, (D) root fungi classes.

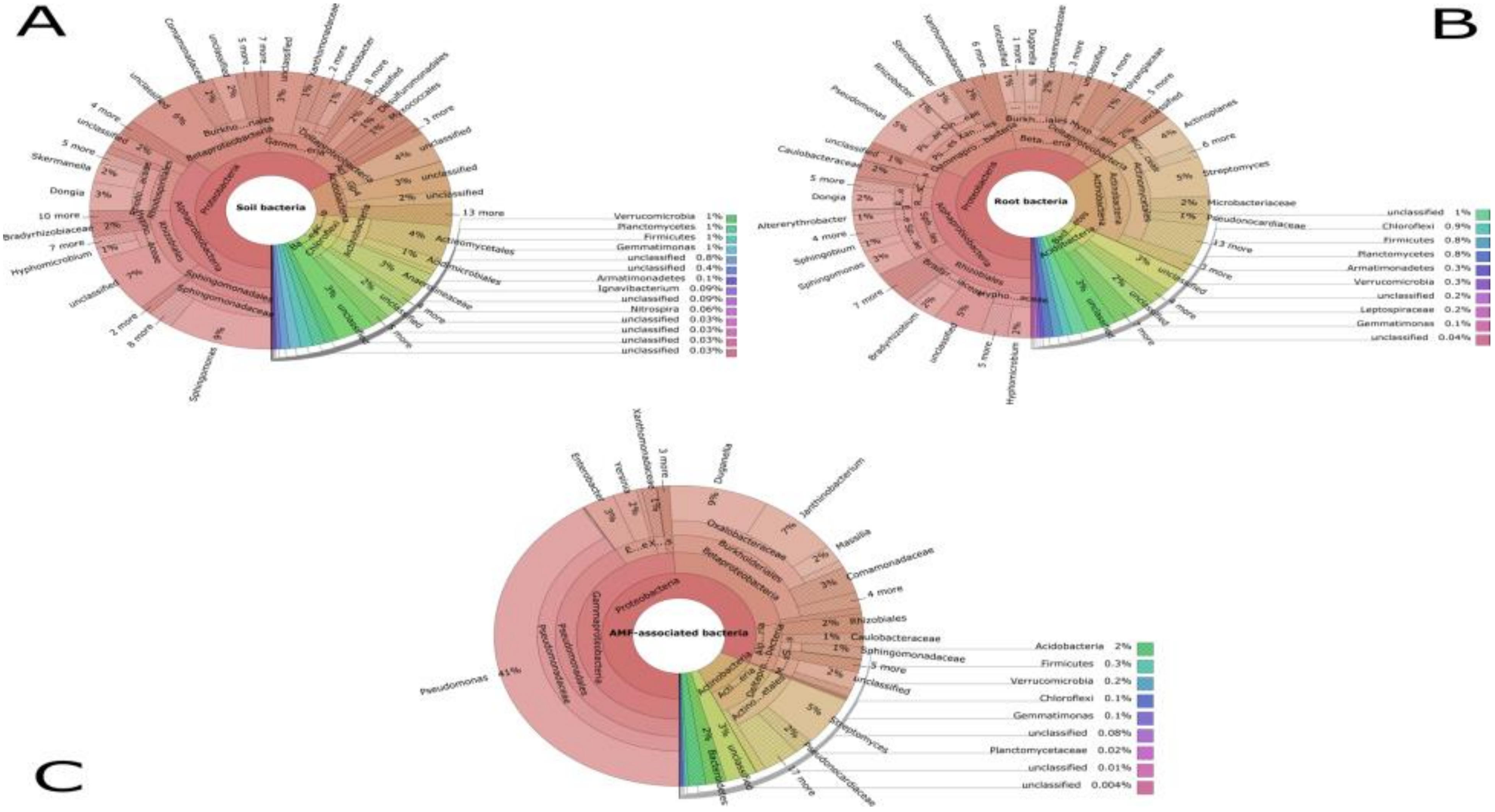

Figure S3. Krona charts showing the taxonomic identification and relative abundance of: (A) rhizosphere soil bacteria, (B) root bacteria and (C) AMF-associated bacteria.

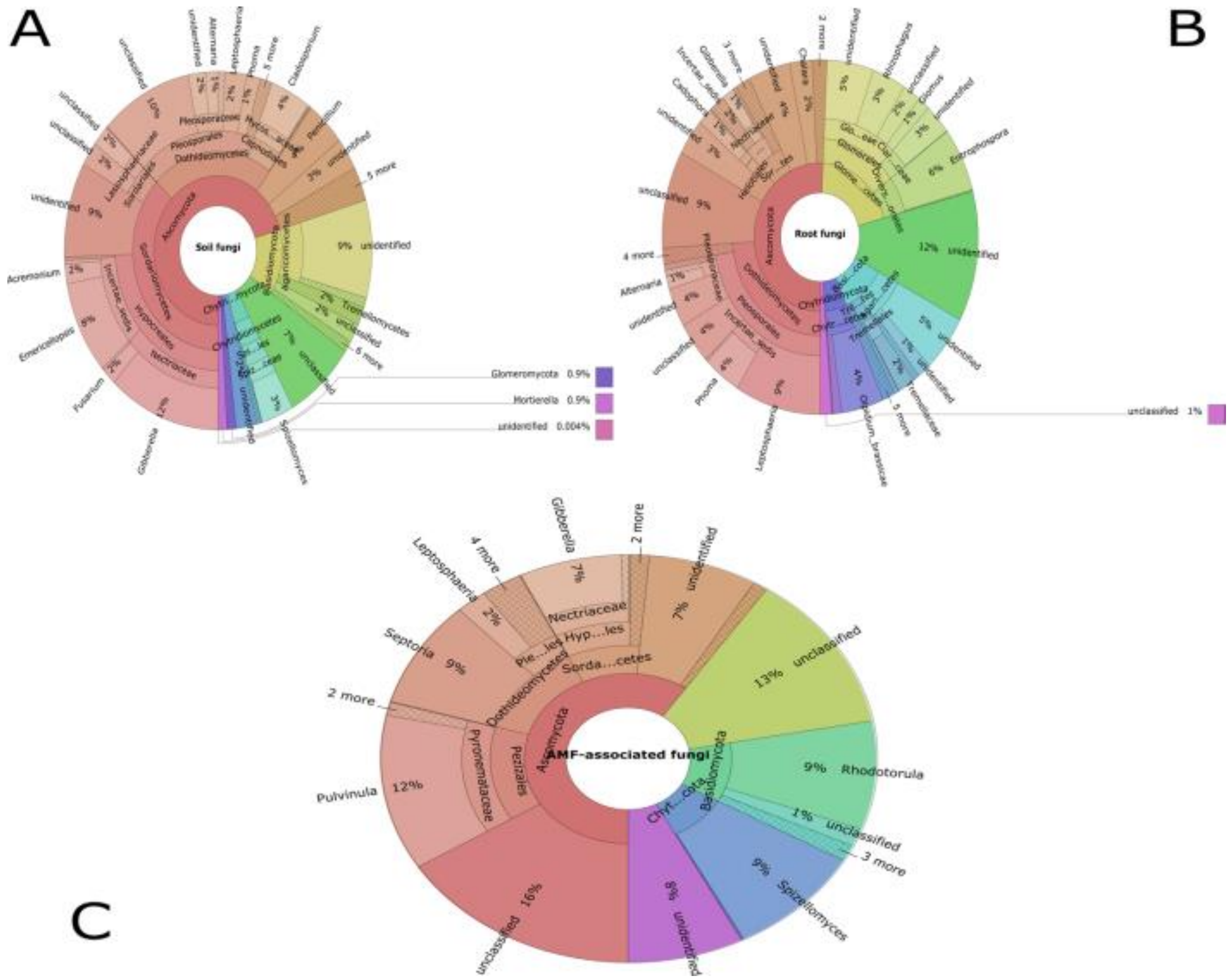

Figure S4. Krona charts showing the taxonomic identification and relative abundance of: (A) rhizosphere soil fungi, (B) root fungi and (C) AMF-associated fungi.

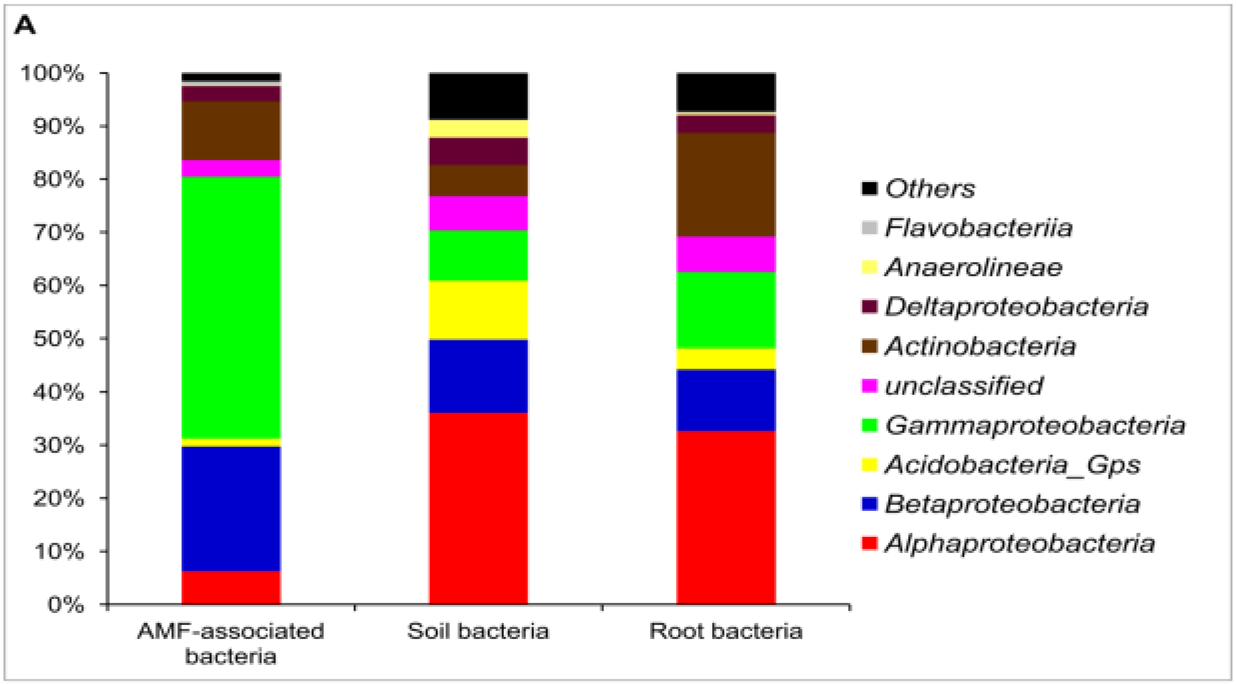

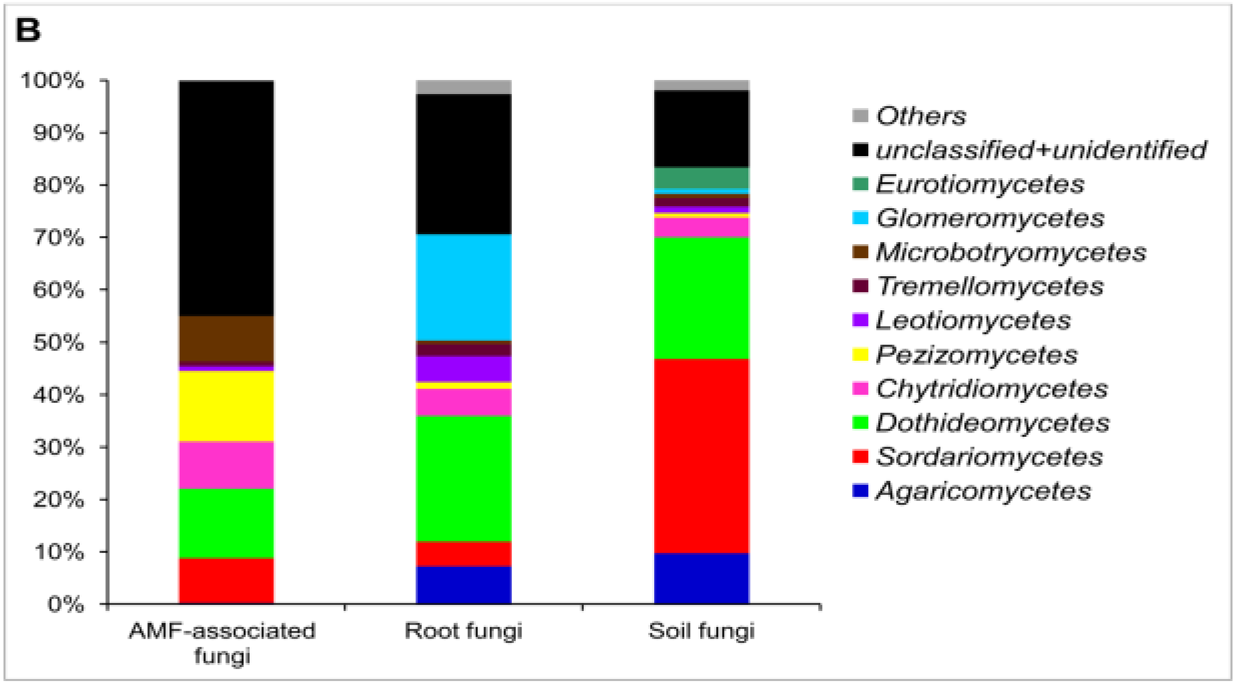

Figure S5. Comparison of relative abundances between: (A) the bacterial classes found in rhizosphere soil, roots and in association with AMF spores; (B) the fungi classes found in rhizosphere soil, roots and in association with AMF spores.
